# Assessment of Anti-Factor Xa Levels in Children Receiving Low-Molecular-Weight Heparin for Treatment and Prophylaxis

**DOI:** 10.3390/children13060792

**Published:** 2026-06-08

**Authors:** Margarita Panova, Maria Spasova, Snezhana Stoencheva, Teodora Dimcheva, Iglika Sotkova-Ivanova, Pamela Boykova

**Affiliations:** 1 Department of Pediatrics “Prof. Dr. Ivan Andreev”, Medical University Plovdiv, 4002 Plovdiv, Bulgaria; maria.spasova@mu-plovdiv.bg (M.S.); pamela.boykova@mu-plovdiv.bg (P.B.); 2 Department of Clinical Laboratory, Medical University Plovdiv, 4002 Plovdiv, Bulgaria; snezhana.stoencheva@mu-plovdiv.bg; 3 Department of Medical Informatics, Biostatistics and E-Learning, Medical University Plovdiv, 4002 Plovdiv, Bulgaria; teodora.dimcheva@mu-plovdiv.bg; 4 Department of Medical Genetics, Medical University Plovdiv, 4002 Plovdiv, Bulgaria; iglika26@abv.bg

**Keywords:** low-molecular-weight heparin, enoxaparin, anti-factor Xa, monitoring, pediatric thrombosis, anticoagulation

## Abstract

**Highlights:**

**What are the main findings?**

- Standard weight-based enoxaparin dosing achieved target anti-Factor Xa levels in only ~57% of pediatric patients, with younger children showing significantly lower levels.
- Age < 1 year, oncological disease, and baseline anti-Xa < 0.3 IU/mL were independent predictors of the need for dose adjustments.

**What are the implications of the main findings?**

- Routine anti-Factor Xa monitoring is essential in pediatric patients to ensure safe and effective LMWH therapy, especially in high-risk groups.
- Current dosing protocols may require age- and disease-specific optimization, particularly in infants and children with oncological conditions.

**Abstract:**

Background: The use of low-molecular-weight heparins (LMWH) for the treatment and prevention of thromboembolic diseases in pediatric patients is increasing, although optimal dosing and monitoring strategies remain insufficiently defined. Methods: This study was conducted at the Clinic of Pediatrics, University Hospital “St. George”, and included 26 hospitalized patients aged 0–18 years with confirmed arterial or venous thrombosis receiving treatment or prophylaxis with enoxaparin. A total of 42 samples were analyzed. Anti-factor Xa activity was measured using an LMWH-calibrated anti-FXa assay (Innovance Heparin, Siemens Healthineers) on a Sysmex CS-2500 analyzer. Therapeutic and prophylactic dosing followed CHEST 2012 guidelines. The study evaluated age- and weight-based dosing, the number of dose adjustments required to achieve target Anti-Xa levels, and the relationship between Anti-Xa levels and anticoagulant response. Results: The mean baseline Anti-Xa level achieved with the initial weight-based dose (1.0 mg/kg/12 h) was significantly lower in children aged 1–2 years compared with older age groups. Older children, as well as patients with oncological and nephrological diseases, achieved higher anticoagulant levels with standard prophylactic dosing. Age under 1 year, oncological disease, and baseline Anti-Xa level < 0.3 IU/mL were identified as independent predictors of the need for more frequent dose adjustments to achieve a therapeutic response. Conclusions: Monitoring of Anti-factor Xa levels is essential in pediatric patients receiving LMWH for both treatment and prophylaxis. Standard dosing regimens may be insufficient in younger children and specific clinical subgroups, supporting the need for individualized dosing strategies.

## 1. Introduction

The incidence of pediatric thromboembolism has increased significantly in recent years [1]. The treatment of thromboembolism—both venous and arterial—is based on the use of unfractionated heparins (UFH), low-molecular-weight heparins (LMWH), and vitamin K antagonists [2,3]. In recent years, direct oral anticoagulants (DOACs), which directly inhibit specific coagulation enzymes, have emerged as a major advancement in pediatric anticoagulant therapy [4]. They are characterized by oral administration, child-friendly formulations, and a substantial reduction in monitoring requirements. They exhibit stable pharmacokinetic and pharmacodynamic profiles, a wide therapeutic window, rapid onset and offset of action, and fewer drug and food interactions [5,6]. However, cumulative clinical experience with their use in children, particularly in those with concomitant chronic diseases, remains limited [7]. Concerns regarding their safety in pediatric practice also persist due to the lack of approved reversal agents for DOACs, as well as limitations associated with therapeutic monitoring [8].

Against this background, the use of low-molecular-weight heparins in childhood for the treatment and prevention of thromboembolic diseases remains widespread [9]. They offer advantages including predictability, stable pharmacokinetics, absence of dietary or drug interactions, reduced risk of heparin-induced thrombocytopenia, and decreased need for monitoring [10]. At the same time, they have a longer half-life, allowing for subcutaneous administration once or twice daily [3]. The pharmacodynamic and pharmacokinetic properties of UFH are complex, resulting in significant interindividual variability in anticoagulant response to the same weight-adjusted dose of UFH [11]. For this reason, monitoring of factor Xa activity is particularly relevant in pediatric clinical practice.

There are differing opinions in the literature regarding routine monitoring of anti-factor Xa (anti-Xa) activity; however, most studies support its necessity [12,13,14,15,16]. Studies evaluating anti-Xa dosing and monitoring in children are limited and often contradictory [15,16]. We considered the unique characteristics of pediatric hemostasis, as well as the complex pharmacodynamic and pharmacokinetic properties of LMWH, which lead to substantial variability in anticoagulant response to weight-adjusted LMWH dosing. Therefore, the aim of the present study was to describe and monitor anti-factor Xa levels in pediatric patients from different age groups receiving therapeutic and prophylactic LMWH treatment and to evaluate its diagnostic reliability in ensuring optimal therapeutic LMWH concentrations.

The objectives of the study were:

1. To determine the therapeutic dose of LMWH (enoxaparin), based on age and body weight, in children with oncological diseases, ischemic stroke, cerebral venous sinus thrombosis, and deep vein thrombosis (DVT).
2. To determine the prophylactic dose of enoxaparin, based on age and body weight, in children with oncological diseases, ischemic stroke, cerebral venous sinus thrombosis, and DVT.
3. To evaluate the number of enoxaparin dose adjustments required to achieve therapeutic anti-factor Xa levels.
4. To assess the relationship between measured anti-Xa levels during enoxaparin therapy and the achieved antithrombotic outcomes.

## 2. Materials and Methods

The study was conducted by a multidisciplinary team including pediatric neurologists and a pediatric oncohematologist at the Clinic of Pediatrics, University Hospital “St. George”, where the initial patient selection was performed. Laboratory analyses of the corresponding parameters were carried out at the Central Clinical Laboratory of University Hospital “St. George”.

A total of 26 children aged 0–18 years, treated at the Clinic of Pediatrics, University Hospital “St. George”, with confirmed arterial or venous thrombosis and receiving either therapeutic treatment or prophylaxis with enoxaparin, were included in the study. Patients with thrombocytopenia or those receiving unfractionated heparins were excluded.

Anti-factor Xa (anti-FXa) activity of low-molecular-weight heparin (LMWH) was measured using an LMWH-calibrated anti-FXa assay (INNOVANCE® Heparin; Siemens Healthineers, Germany) performed on a Sysmex CS-2500 automated coagulation analyzer (Sysmex Corporation, Kobe, Japan) according to the manufacturer’s instructions.

Patients included in the study received an initial therapeutic dose of 1.5 mg/kg for patients younger than 2 months of age and 1.0 mg/kg for patients older than 2 months of age. The dose was administered subcutaneously every 12 h in order to achieve anti-factor Xa (anti-Xa) levels between 0.5 and 1.0 IU/mL, measured from a blood sample collected 4 to 6 h after subcutaneous injection [3]. Peak anti-Xa levels were assessed after the third LMWH dose.

Once therapeutic serum anti-Xa levels were achieved, no subsequent anti-Xa measurements were performed unless clinically indicated.

If therapeutic serum anti-Xa levels were not achieved, dose adjustments were performed according to the nomogram provided in the CHEST guidelines [3] until therapeutic serum anti-Xa levels were reached.

For prophylaxis with LMWH, doses of 0.75 mg/kg were administered to infants younger than 12 months of age and 0.5 mg/kg per dose to older children above 12 months of age. Administration was again performed every 12 h. The dose was adjusted to achieve target anti-factor Xa levels of 0.2–0.4/0.5 IU/mL.

Anti-Xa levels were measured 4 to 6 h after the third dose from treatment initiation and following each dose adjustment. If the level was below 0.2 IU/mL, the dose was increased by 20%. If the level was between 0.2 and 0.5 IU/mL, no dose adjustment was performed. If the level was between 0.51 and 0.7 IU/mL, the dose was reduced by 20%. If the level was between 0.71 and 1.0 IU/mL, the dose was reduced by 30%. If the level exceeded 1.0 IU/mL, one dose was withheld.

## 3. Results

### 3.1. Statistical Methods

Categorical variables were presented as frequencies (n) and percentages (%), whereas continuous variables were expressed as mean ± standard deviation (SD) or median with interquartile range (IQR), depending on the normality of distribution. Normality was assessed using the Shapiro–Wilk test. The results showed that all investigated variables, except baseline Anti-Xa level (IU/mL) (W = 0.925, *p* = 0.060), deviated significantly from normal distribution (*p* < 0.05).

Comparison of baseline Anti-Xa levels between age groups was performed using one-way analysis of variance (One-way ANOVA) with Tukey post hoc test for multiple comparisons. The relationship between age and Anti-Xa levels was assessed using Pearson correlation analysis. Comparison of Anti-Xa levels between diagnostic groups was performed using the nonparametric Kruskal–Wallis test, followed by Dunn’s test with Bonferroni correction for post hoc analysis.

The distribution of patients according to achievement of the target prophylactic range was analyzed using the chi-square test. Differences in prophylactic Anti-Xa levels between age groups were evaluated using One-way ANOVA.

The number of required dose adjustments was modeled using negative binomial regression due to the presence of overdispersion in the data. Factors associated with the need for more frequent dose adjustments were identified through multivariable regression analysis. The effectiveness of each adjustment was evaluated using repeated measures ANOVA.

Time in therapeutic range (TTR) was calculated using the linear interpolation method of Rosendaal et al. [11]. TTR was defined as the percentage of total treatment time during which interpolated daily Anti-Xa values remained within the target therapeutic range of 0.5–1.0 IU/mL. The relationship between TTR and the stability of anticoagulant effect was assessed using Spearman correlation analysis. Due to the absence of clinical events, the analysis focused on pharmacodynamic response.

The stability of anticoagulant effect was assessed by calculating the coefficient of variation of follow-up Anti-Xa measurements for each patient. Differences in stability between diagnostic and age groups were analyzed using One-Way ANOVA.

To control for potential confounding factors, multivariable linear regression models were applied for continuous outcomes (Anti-Xa levels, TTR), and multivariable Poisson regression models were used for the number of dose adjustments. Collinearity between independent variables was assessed using the variance inflation factor (VIF), with values above 5 considered indicative of significant collinearity.

Sensitivity analyses were performed to assess the robustness of the results by excluding patients with extreme values, defined using the interquartile range (IQR) method.

Additionally, to account for correlations between repeated measurements within the same patient, linear mixed-effects models were applied. The models included fixed effects for age group and time of measurement, as well as a random effect at the patient level (random intercept per patient). To model temporal dependence between repeated observations, a first-order autoregressive covariance structure (AR(1)) was selected.

The proportion of missing data was below 5% for all analyzed variables. Missing data were handled using the Full Information Maximum Likelihood approach.

To assess sample size power (n = 26 patients, 45 observations), a post hoc power analysis was performed based on ANOVA comparing Anti-Xa levels between age groups. The significance level was set at α = 0.05, with an expected effect size of f = 0.25 (medium, based on clinical relevance and treatment protocols described by Monagle et al. [3]) and number of groups = 4.

All statistical analyses were performed using IBM SPSS Statistics version 26.0 (IBM Corp., Armonk, NY, USA). Statistical significance was defined as *p* < 0.05 (Table 1).

### 3.2. Objective 1

Fourteen pediatric patients were included in the study (mean age 10.2 ± 5.8 years; 8 boys (57.1%) and 6 girls (42.9%)) receiving therapeutic doses of enoxaparin for confirmed arterial and venous thromboses. The distribution of the main thrombotic events was as follows: ischemic stroke (n = 6, 42.9%), cerebral venous sinus thrombosis (n = 5, 35.7%), deep vein thrombosis (n = 2, 14.3%), and non-cerebral arterial thrombosis (n = 1, 7.1%) (Table 2).

The initial weight-based dose (1.0 mg/kg/12 h) resulted in therapeutic anti-Xa levels (0.5–1.0 IU/mL) after the first administration in eight patients (57.1%), who did not require subsequent dose adjustment. In the remaining six patients (42.9%), the initial anti-Xa level was subtherapeutic, necessitating further dose titration according to the CHEST protocol [3].

The measured mean baseline Anti-Xa level was 0.41 ± 0.26 IU/mL. One-way ANOVA demonstrated significant differences in baseline Anti-Xa levels between age groups (F = 4.32; *p* = 0.016). Post hoc analysis using Tukey’s test identified significantly lower levels in children aged 1 to 2 years (0.28 ± 0.21 IU/mL) compared with children older than 10 years (0.45 ± 0.27 IU/mL; *p* = 0.032).

The Kruskal–Wallis test revealed significant differences in Anti-Xa levels between diagnostic groups (χ^2^ = 15.73; *p* = 0.015). Dunn–Bonferroni post hoc testing showed significantly lower levels in patients with oncological diseases (0.25 ± 0.18 IU/mL) compared with those with deep vein thrombosis (0.49 ± 0.29 IU/mL; *p* = 0.028). The results are presented in Table 3.

The calculated Pearson’s correlation coefficient demonstrated a moderate negative correlation between age and Anti-Xa levels (r = −0.42; *p* = 0.038), as well as a weak positive correlation between body weight and Anti-Xa levels (r = 0.31; *p* = 0.127).

### 3.3. Objective 2

The prophylactic treatment group included 12 patients with a mean age of 9.8 ± 6.2 years, of whom 4 (33.3%) were boys and 8 (66.7%) were girls. The distribution by diagnostic groups was as follows: nephrological diseases 3 (42.9%), oncohematological diseases 5 (28.6%), and other diseases 4 (7.1%) (Table 4).

Only 33.3% of patients in the prophylactic treatment group achieved the target range (0.2–0.5 IU/mL). Fisher’s exact test did not demonstrate a statistically significant difference in achieving the target range between the four age groups (*p* = 0.089).

The nonparametric Kruskal–Wallis test demonstrated significant differences in Anti-Xa levels between age groups (H = 8.47; *p* = 0.037). Dunn–Bonferroni post hoc analysis showed that levels in children under 1 year of age (0.12 ± 0.04 IU/mL) were significantly lower compared with those in children older than 10 years (0.44 ± 0.16 IU/mL; *p* < 0.05) (Table 5).

Linear regression analysis, adjusted for age, showed that the presence of conditions associated with high thrombotic risk (oncological and nephrological diseases) was independently associated with higher Anti-Xa levels after standard prophylactic dosing (β = 0.16; 95% CI: 0.04–0.28; *p* = 0.016). The regression model explained 68% of the variation in Anti-Xa levels (R^2^ = 0.68, *p* = 0.003).

A significant positive correlation between age and Anti-Xa levels was identified (ρ = 0.62; *p* = 0.032), indicating that older children achieved higher anticoagulant levels with standard prophylactic dosing.

For proportions, the Clopper–Pearson exact method was used. For means and mean differences, t-based confidence intervals were applied.

Due to the small size of some groups and the nonparametric distribution of the data, comparisons between age groups were performed using the Kruskal–Wallis test (H) with Dunn–Bonferroni post hoc analysis.

### 3.4. Objective 3

The mean number of dose adjustments was 2.4 ± 1.7 (range: 0–6). Negative binomial regression identified three independent factors associated with the need for more frequent dose adjustments: age under 1 year (IRR = 1.89; 95% CI: 1.32–2.71; *p* = 0.008), oncological disease (IRR = 1.67; 95% CI: 1.15–2.42; *p* = 0.017), and baseline Anti-Xa level < 0.3 IU/mL (IRR = 2.12; 95% CI: 1.48–3.04; *p* = 0.001) (Table 6).

Repeated measures ANOVA showed that each dose adjustment resulted in a mean increase of 0.18 IU/mL in Anti-Xa levels (F = 28.43; *p* < 0.001). The effect was more pronounced in patients with initially low levels (<0.3 IU/mL), where each adjustment led to an increase of 0.24 IU/mL.

Statistical significance for factors influencing the number of dose adjustments was assessed using negative binomial regression; the effect of dose adjustments on Anti-Xa levels was evaluated using repeated measures ANOVA; and correlations were analyzed using Spearman’s correlation test.

**Figure 1:**
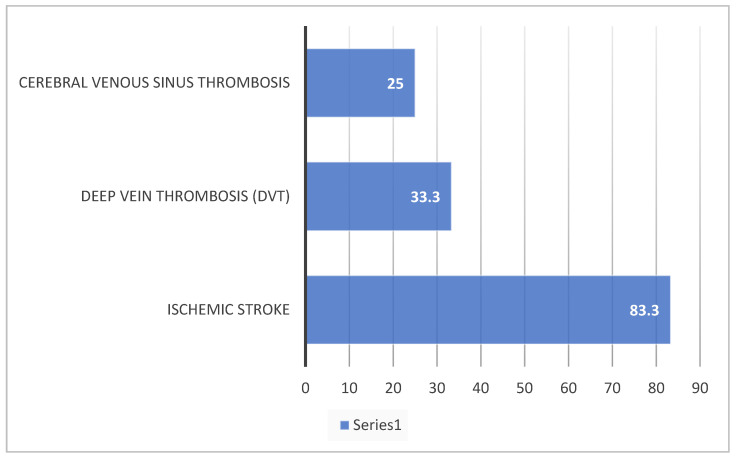
Distribution of patients requiring dose titration by diagnostic groups (%).

The need for dose titration was also diagnosis-dependent. A total of 83.3% (n = 5) of patients with ischemic arterial stroke required dose adjustment, compared with 25.0% (n = 2) of patients with cerebral venous sinus thrombosis or deep vein thrombosis (Figure 1).

Comparative analysis between patients requiring dose adjustment (n = 7) and those with stable dosing (n = 7) revealed significant differences. Patients in the adjustment group had significantly lower baseline Anti-Xa levels (0.23 ± 0.11 IU/mL vs. 0.66 ± 0.27 IU/mL; *p* = 0.009, Mann–Whitney U test) and were significantly younger (7.7 ± 5.8 years vs. 12.1 ± 5.0 years; *p* = 0.027, independent samples *t*-test). A baseline Anti-Xa level below 0.35 IU/mL was identified as a strong predictor of the need for subsequent dose adjustment.

The quality of anticoagulation, measured by time in therapeutic range (TTR), was evaluated in patients (n = 7) who required dose adjustment. The mean TTR for all patients was 58.2 ± 33.5%. A significant difference in TTR was observed between the study groups. Patients without dose modification during treatment achieved excellent anticoagulation control, with a mean TTR of 85.0%, compared with patients undergoing dose titration, who had a significantly lower mean TTR of 27.4% (*p* = 0.029), reflecting the initial period of instability before achieving a stable therapeutic state (Table 7).

### 3.5. Objective 4

The mean time in therapeutic range (TTR) was 32.2% ± 22.1%. Spearman’s correlation coefficient demonstrated a significant positive correlation between TTR and the stability of the anticoagulant effect (ρ = 0.68; *p* < 0.001). Patients with TTR > 40% (n = 6; 23.1%) showed a significantly lower coefficient of variation of follow-up Anti-Xa measurements (18.2% vs. 42.7%; *p* < 0.001).

One-way ANOVA revealed significant differences in therapy stability between diagnostic groups (F = 3.84; *p* = 0.012). Patients with deep vein thrombosis demonstrated the highest stability (coefficient of variation = 15.3%), whereas patients with oncological diseases showed the lowest stability (coefficient of variation = 47.8%) (Table 8).

The multivariable linear regression model for TTR identified three independent predictors: age over 10 years (β = 0.51, *p* = 0.004), absence of oncological disease (β = 0.43, *p* = 0.018), and a lower number of dose adjustments (β = −0.39, *p* = 0.028). The model explained 62% of the variation in TTR (R^2^ = 0.62, *p* < 0.001) (Table 9).

The multivariable Poisson regression model confirmed that age under 1 year (IRR = 1.76, 95% CI: 1.21–2.56, *p* = 0.012), oncological disease (IRR = 1.58, 95% CI: 1.09–2.29, *p* = 0.024), and low baseline Anti-Xa level (IRR = 1.94, 95% CI: 1.35–2.78, *p* = 0.002) remained significant predictors of the need for more frequent dose adjustments after adjustment for potential confounding factors.

### 3.6. Additional Analyses

The post hoc power analysis demonstrated that, assuming an effect size of f = 0.25 and a significance level of α = 0.05, the study had 75% statistical power to detect significant differences in Anti-Xa levels between age groups. This result indicates moderate statistical power, sufficient for identifying medium-sized effects.

Sensitivity analysis identified three extreme values (0.95 IU/mL, 0.91 IU/mL, and 0.84 IU/mL), which were excluded from subsequent calculations. After removal of these outliers, the results of the ANOVA analysis for age groups remained significant (*p* = 0.045, compared with the initial *p* = 0.019). The mean Anti-Xa level in the adjusted dataset was 0.40 IU/mL (SD = 0.19), comparable to the initially calculated mean value of 0.43 IU/mL (SD = 0.22). This consistency supports the robustness of our main findings.

Application of linear mixed-effects models, accounting for repeated measurements, confirmed the significance of the effect of age group on Anti-Xa levels (*p* = 0.03). The measurement time point did not have a significant effect (*p* = 0.25). Comparative analysis with ordinary linear models showed minimal differences in parameter estimates, while the mixed-effects model demonstrated better fit to the data (AIC = −50.2 vs. AIC = −45.1). These results further strengthen the reliability of the conclusions despite the limitations of the sample size.

* AIC (Akaike Information Criterion) is a measure of the quality of a statistical model.

## 4. Discussion

The initial weight-based dose (1.0 mg/kg/12 h) administered to patients in our study resulted in therapeutic anti-factor Xa levels (0.5–1.0 IU/mL) in 57.1% of the treatment group receiving LMWH after the initial administration. In the remaining six patients (42.9%), baseline anti-Xa levels were subtherapeutic, requiring subsequent dose titration according to the CHEST protocol [3]. Our findings are comparable to those reported by Leung et al. [17], in which 62% of patients achieved anti-Xa levels within the target range. Similar results have also been reported in other studies [18,19,20,21,22,23,24,25,26,27,28,29]. Massicotte et al. found that only 50% (10/20) of their patients achieved therapeutic anti-Xa levels after receiving a dose of 1 mg/kg administered subcutaneously every 12 h [18]. A higher percentage (68%) of patients reaching therapeutic levels was reported in the study by Obuljen Jasna [15]. The measured anti-FXa activity (median 0.65 IU/mL) in that study was comparable to our observed value of 0.41 ± 0.26 IU/mL [15]. Unlike the results reported in that study and in the study by Klaassen et al. [21], no major hemorrhagic complications or recurrent venous thromboembolism were observed among our patients. Our study confirms the conclusion of Klaassen et al. [21] that LMWH is safe and effective for both therapeutic and prophylactic treatment of venous thromboembolism (VTE) in children.

Our results demonstrated significant differences in baseline anti-Xa levels between age groups and identified significantly lower levels in children aged 1–2 years compared with children older than 10 years. We also observed a moderate negative correlation between age and anti-Xa levels, consistent with the findings reported by Klaassen et al. [21]. They reported the need for higher initial therapeutic doses of LMWH compared with recommended doses to achieve target ranges, particularly in neonates and children younger than 5 years. Comparable age-related variability in anti-Xa response has consistently been documented in neonates and infants. Greene et al. emphasized that neonates and young infants frequently require higher weight-based doses of low-molecular-weight heparin to achieve target anti-Xa concentrations [22]. Similarly, Ignjatovic et al. reported substantial interindividual variability in anti-Xa response among neonates and infants, with a considerable proportion of patients failing to achieve therapeutic ranges despite protocol-based dosing regimens [23].

Neonates and young infants require higher doses of low-molecular-weight heparin (LMWH) to achieve therapeutic levels compared with older children due to lower antithrombin levels and more rapid elimination of the drug [24].

We also identified differences in anti-Xa levels among the different etiological groups. Patients with oncological diseases demonstrated lower anti-Xa levels compared with those with deep vein thrombosis. Children with malignancies often exhibit a larger volume of distribution and more rapid clearance of low-molecular-weight heparin. Consequently, they frequently require higher doses to achieve target anti-Xa levels. At the same time, pediatric oncology patients (particularly those receiving L-asparaginase treatment) may present with reduced antithrombin levels, which are necessary for LMWH to exert its anticoagulant effect [25].

Only 33.3% of patients in our prophylaxis group achieved the target range (0.2–0.5 IU/mL). The mean Anti-Xa level measured in our study (0.35 ± 0.18 IU/mL) was comparable to the values reported by Obuljen Jasna [15], who observed median Anti-Xa levels of 0.27 IU/mL ranging from 0.10 to 0.78 IU/mL. We identified significant differences in anti-Xa levels among children receiving prophylactic doses of enoxaparin across age groups. Levels in children younger than one year were significantly lower than those observed in children older than 10 years, consistent with our findings in children receiving therapeutic LMWH treatment.

In our study, the presence of conditions associated with high thrombotic risk (oncological and nephrological diseases) was independently associated with higher anti-Xa levels following administration of standard prophylactic doses. Similar findings were reported by Marshall et al. [14] in critically ill pediatric patients admitted to intensive care units. Their observations suggested that more than 20% of patients receiving prophylactic enoxaparin for VTE exhibited elevated serum anti-Xa levels corresponding to true therapeutic anticoagulation. We also identified a significant positive correlation between age and anti-Xa levels, confirming that older children achieve higher anticoagulant levels under standard prophylactic dosing. The results of our study further support the need for anti-Xa monitoring during prophylactic administration of LMWH.

We observed a mean number of dose adjustments of 2.4 ± 1.7 (range: 0–6), which is comparable to the results reported by Andrade-Campos et al. [26], who described an average of three dose modifications and 11 days required to achieve target anti-Xa levels. In the study by Ho et al. [27], the number of required dose adjustments per patient ranged from 1 to 11, representing a wider range than observed in our cohort. These findings again suggest the necessity of routine anti-Xa monitoring.

We found that each dose adjustment resulted in an average increase of 0.18 IU/mL in anti-Xa levels, with a more pronounced effect among patients with initially low levels (<0.3 IU/mL), where each adjustment resulted in an increase of 0.24 IU/mL. We did not identify similarly detailed characterization of dose adjustment stages in pediatric enoxaparin treatment within the available literature.

We identified three independent factors associated with the need for more frequent dose adjustments: age below one year, oncological disease, and baseline anti-Xa level < 0.3 IU/mL. Leung et al. [17] also identified age as a factor associated with more frequent dose adjustments, although their study focused on patients ≤ 2 months of age. In 2021, Koury et al. [16] reported an increased need for enoxaparin dose modifications through anti-Xa monitoring in patients with venous thromboembolism younger than six months of age [10]. Our findings support both studies regarding the relationship between age and number of dose adjustments but extend this association to patients younger than one year. We additionally identify oncological disease as a factor alongside previously recognized factors reported by Leung et al. [17] and Koury et al. [16], including cardiac disease, renal insufficiency, and intensive care hospitalization, as being more frequently associated with dose adjustment.

The association between low baseline anti-Xa levels (<0.3 IU/mL) and the requirement for more frequent dose adjustments is biologically plausible. Children younger than one year exhibit a larger volume of distribution, faster renal clearance, and differences in plasma protein binding compared with older children and adults. This makes LMWH pharmacokinetics less predictable and requires more frequent dose adjustment to achieve target anti-Xa levels [24].

Cancer-associated thrombosis creates a hypercoagulable state and alters drug metabolism. Malignancy and its treatment (e.g., asparaginase, corticosteroids) further modify hemostasis. Pediatric oncology patients frequently present with fluctuating platelet counts and impaired hepatic and renal function, rendering fixed-dose LMWH regimens less predictable [25]. Greater fluctuations in anti-Xa activity are also more common due to altered pharmacokinetics, including increased volume of distribution and accelerated enoxaparin clearance. This variability often results in subtherapeutic levels and a greater need for frequent dose adjustments to achieve target therapeutic ranges [28,29].

We assessed the quality of anticoagulation in our patients using time in therapeutic range (TTR). Patients who required no dose adjustment during treatment achieved excellent anticoagulation control with a mean TTR of 85.0%, whereas patients undergoing dose titration demonstrated a significantly lower mean TTR of 27.4%, reflecting the initial instability period before reaching a stable therapeutic state. To our knowledge, no studies have evaluated anticoagulation quality using TTR in this context.

We also observed differences in the stability of anticoagulant response across different nosological groups. Again, patients with oncological diseases demonstrated the lowest stability of anticoagulant response, whereas patients with deep vein thrombosis exhibited the highest response stability. This phenomenon is likely multifactorial, reflecting altered pharmacokinetic and pharmacodynamic profiles secondary to systemic inflammation, hypoalbuminemia, fluctuations in renal and hepatic function, and concomitant oncological therapy that modulates coagulation homeostasis. In addition, oncological treatment may cause drug interactions that alter anticoagulant metabolism and anticoagulant response [28]. We did not identify studies specifically evaluating differences in anticoagulant response stability among different patient groups.

### Limitations

Several limitations of the present study warrant explicit acknowledgment, including the relatively small sample size and the lack of standardization of anti-Xa monitoring.

The relatively modest sample size inherently limits the statistical power of the analyses and increases the susceptibility of multivariable models to overfitting. Although we deliberately restricted the number of covariates to a limited set of a priori defined, clinically and biologically plausible predictors, and although negative binomial regression was employed to appropriately account for overdispersion in the count data, the resulting estimates should nevertheless be interpreted with appropriate caution. Accordingly, the present findings are best regarded as exploratory and hypothesis-generating, providing a rational basis for subsequent confirmatory investigations.

Future prospective, adequately powered, multicenter studies incorporating clinically meaningful endpoints—including thrombotic recurrence, bleeding complications, and long-term outcomes—are warranted to validate the present observations and to more precisely define the role of anti-factor Xa monitoring in optimizing low-molecular-weight heparin therapy in the pediatric population.

## 5. Conclusions

The present study demonstrated that the mean baseline anti-factor Xa level achieved using the initial weight-based dosing regimen (1.0 mg/kg/12 h) was significantly lower in children aged 1–2 years, indicating age-related differences in anticoagulant response.

Our findings suggest that pediatric patients require higher LMWH doses at younger ages, with dose requirements progressively decreasing as age increases.

Older children, as well as patients with oncological and nephrological diseases, achieved higher anticoagulant levels under standard prophylactic dosing, highlighting the influence of both age and underlying disease on anti-Xa response.

Furthermore, age below 1 year, oncological disease, and baseline anti-Xa levels below 0.3 IU/mL were identified as independent factors associated with the need for more frequent dose adjustments to achieve a therapeutic response.

Overall, these results support the importance of individualized LMWH dosing and routine anti-factor Xa monitoring in pediatric patients receiving both therapeutic and prophylactic anticoagulation in order to optimize treatment efficacy and safety.

## Figures and Tables

**Table 1:** Descriptive statistics of the study cohort of 26 children.

Parameter	Number (n)	%
Sex		
Boys	12	46.15
Girls	14	53.85
Age		
Range	From 1 month to 18 years.	
Mean ± SEM	10.44 ± 1.19	
Median (IQR)	12.50 (12.00)	
Boys		
Range	From 1 year and 10 months to 18 years.	
Mean ± SEM	10.32 ± 1.96	
Median (IQR)	11.50 (14.50)	
Girls		
Range	From 1 month to 16 years.	
Mean (±SEM)	10.54 ± 1.51	
Median (IQR)	12.50 (15.92)	
Diagnosis—groups		
✓ Cerebral venous sinus thrombosis	5	19.23
✓ Cerebellar or cerebral arterial thrombosis/ischemic stroke	6	23.08
✓ Non-cerebral arterial thrombosis	1	3.85
✓ Deep vein thrombosis (DVT)	2	7.69
✓ Nephrological disease	3	11.54
✓ Oncohematological diseases	5	19.23
✓ Other diseases	4	15.38
Type of treatment		
✓ Therapeutic treatment	14	53.85
✓ Prophylaxis	12	46.15

Due to the wide variability in the age range, it is more appropriate to report the median and interquartile range (IQR) rather than the mean ± standard error of the mean (SEM).

**Table 2:** Characteristics of patients receiving therapeutic treatment (n = 14).

Characteristics	Value
Age (Mean ± SD)	10.2 ± 5.8
Sex male/female (n, %)	8 (57.1%)/6 (42.9%)
Age group according to dosing, n (%)	
1–2 years	2 (14.3%)
2–10 years	4 (28.6%)
>10 years	8 (57.1%)
By diagnostic groups, n (%)	
Ischemic stroke	6 (42.9%)
Cerebral venous sinus thrombosis	5 (35.7%)
Non-cerebral arterial thrombosis	1 (7.1%)
Deep vein thrombosis (DVT)	2 (14.3%)

**Table 3:** Results of therapeutic enoxaparin dosing analysis (n = 14).

Parameters	Anti-Xa Level (IU/mL)	95% CI (U/mL)	*p*-Value
Baseline Anti-Xa level (IU/mL)	0.41 ± 0.26	0.309–0.519	
By age groups			
1–2 years	0.35 ± 0.19	−0.306–0.932	0.016 ^1^
2–10 years	0.39 ± 0.23	0.185–0.481	
>10 years	0.45 ± 0.27	0.362–0.684	
By diagnostic groups:			
Ischemic stroke	0.51 ± 0.30	0.188–0.840	0.015 ^2^
Cerebral venous sinus thrombosis	0.42 ± 0.18	0.276–0.572	
Non-cerebral arterial thrombosis	0.25	N/A	
Deep vein thrombosis (DVT)	0.28 ± 0.31	−0.424–0.984	

Data are presented as mean ± SD; 95% confidence interval (95% CI); ^1^ one-way ANOVA; ^2^ Kruskal–Wallis test.

**Table 4:** Characteristics of patients in the prophylactic group (n = 12).

Characteristics	Value
Age (Mean ± SD)	9.8 ± 6.2
Sex male/female (n, %)	4 (33.3%)/8 (66.7%)
Age group according to dosing, n (%)	
<2 months	1 (8.3%)
≥2 months	11 (91.7%)
By diagnostic groups, n (%)	
Nephrological disease	3 (42.9%)
Oncohematological diseases	5 (28.6%)
Other diseases	4 (7.1%)

**Table 5:** Analysis of prophylactic enoxaparin dosing (n = 12).

Parameters	Anti-Xa Level (IU/mL)	95% CI (U/mL)	*p*-Value
Achievement of target range (0.2–0.5 IU/mL) (n = 4)	33.3%	9.9–65.1%	
Mean Anti-Xa level (IU/mL)	0.35 ± 0.18	0.23–0.47	
Comparison of Anti-Xa levels between age groups			
<1 year	0.12 ± 0.04	0.03–0.20	0.037
1–2 years	0.27	N/A	
2–10 years	0.27 ± 0.06	−0.28–0.81	
>10 years	0.44 ± 0.16	0.28–0.59	
Post hoc analysis (comparison with the >10 years group)			
<1 year vs. >10 years			0.046
2–10 years vs. >10 years			0.215

Data are presented as n (%) and mean ± SD; 95% confidence interval (95% CI).

**Table 6:** Results of the analysis of dose adjustments and therapy stability.

Parameters	Value	95% CI	*p*-Value
Number of dose adjustments	2.4 ± 1.7	1.7–3.1	
Factors associated with more frequent dose adjustments:			
Age < 1 year	IRR = 1.89	1.32–2.71	0.008
Oncological disease	IRR = 1.67	1.15–2.42	0.017
Anti-Xa < 0.3 IU/mL	IRR = 2.12	1.48–3.04	0.001
Effect of dose adjustment	+0.18 U/mL	0.12–0.24	<0.001
Time in therapeutic range (TTR)	32.2% ± 22.1%	23.1–41.3%	
Correlation between TTR and stability	ρ (rho) = 0.68	0.45–0.83	<0.001
Coefficient of variation (CV):			
TTR > 40%	18.2%	14.1–22.3%	<0.001
TTR < 40%	42.7%	35.8–49.6%	

Data are presented as mean ± standard deviation or 95% confidence interval (95% CI); Incidence Rate Ratio (IRR); Time in Therapeutic Range (TTR); ρ (rho): Spearman’s correlation coefficient.

**Table 7:** Comparative analysis of patients receiving treatment (with and without enoxaparin dose titration).

Parameters	Patients Without Dose Titration (n = 7)	Patients with Dose Titration (n = 7)	*p*-Value
Age, years (Mean ± SD)	12.1 ± 5.0	7.7 ± 5.8	0.027 ^1^
Baseline Anti-Xa level, IU/mL (Mean ± SD)	0.66 ± 0.27	0.23 ± 0.11	0.009 ^2^
Number of dose adjustments (Mean ± SD)	-	2.8 ± 1.7	N/A
Time in therapeutic range (TTR, %)	85.0 ± 24.2 (n = 3)	27.4 ± 21.6 (n = 4)	0.029 ^1^

Data are presented as mean ± standard deviation (Mean ± SD); Time in Therapeutic Range (TTR); ^1^ independent samples *t*-test; ^2^ Mann–Whitney U test.

**Table 8:** Comparison of therapy stability between diagnostic groups.

Diagnostic Group	Coefficient of Variation (%)	95% CI	*p*-Value
Deep vein thrombosis (DVT)	15.3	10.8–19.8	reference
Ischemic stroke	24.7	18.9–30.5	0.043
Cerebral venous sinus thrombosis	28.9	21.4–36.4	0.018
Oncohematological diseases	47.8	38.2–57.4	<0.001
Nephrological diseases	31.2	22.8–39.6	0.026
Other diseases	35.6	27.1–44.1	0.009

Data are presented as coefficient of variation (CV) and 95% confidence interval (95% CI). One-way ANOVA was applied to assess whether statistically significant differences existed between the mean values.

**Table 9:** Multivariable regression analysis of predictors of TTR and number of dose adjustments.

Predictor	Coefficient (β)	IRR	95% CI	*p*-Value
For TTR (R^2^ = 0.62)				
Age > 10 years	0.51		0.28–0.74	0.004
Absence of oncological disease	0.43		0.18–0.68	0.018
Fewer dose adjustments	−0.39		−0.62–−0.16	0.028
For number of dose adjustments:				
Age < 1 year		1.76	1.21–2.56	0.012
Oncological disease		1.58	1.09–2.29	0.024
Anti-Xa < 0.3 IU/mL		1.94	1.35–2.78	0.002

Data are presented as Time in Therapeutic Range (TTR); standardized regression coefficient (β), indicating the direction and strength of the association; 95% confidence interval (95% CI); and Incidence Rate Ratio (IRR).

## Data Availability

The data presented in this study are available on reasonable request from the corresponding author. The data are not publicly available because the study involves a small cohort of pediatric patients, and public deposition of the dataset could compromise participant confidentiality and increase the risk of indirect identification. Data sharing is therefore restricted in accordance with ethical approval requirements and institutional policies regarding the protection of personal health information.

## Abbreviations

The following abbreviations are used in this manuscript:

LMWH	Low-Molecular-Weight Heparin
Anti-Xa	Anti-Factor Xa
DVT	Deep Vein Thrombosis
TTR	Time in Therapeutic Range
CV	Coefficient of Variation
IQR	Interquartile Range
SD	Standard Deviation
SEM	Standard Error of the Mean
ANOVA	Analysis of Variance
VIF	Variance Inflation Factor
IRR	Incidence Rate Ratio
CI	Confidence Interval
NTC	Non-Template Control

## References

[1] Raffini L., Huang Y.S., Witmer C., Feudtner C. (2009). Dramatic increase in venous thromboembolism in children’s hospitals in the United States from 2001 to 2007. Pediatrics.

[2] Bohnhoff J.C., DiSilvio S.A., Aneja R.K., Shenk J.R., Domnina Y.A., Brozanski B.S., Good M. (2017). Treatment and follow-up of venous thrombosis in the neonatal intensive care unit: A retrospective study. J. Perinatol..

[3] Monagle P., Chan A.K.C., Goldenberg N.A., Ichord R.N., Journeycake J.M., Nowak-Göttl U., Vesely S.K. (2012). Antithrombotic therapy in neonates and children: Antithrombotic Therapy and Prevention of Thrombosis, 9th ed: American College of Chest Physicians Evidence-Based Clinical Practice Guidelines. Chest.

[4] Roehrig S., Straub A., Pohlmann J., Lampe T., Pernerstorfer J., Schlemmer K.H., Reinemer P., Perzborn E. (2005). Discovery of the novel antithrombotic agent 5-chloro-N-({(5S)-2-oxo-3- [4-(3-oxomorpholin-4-yl)phenyl]-1,3-oxazolidin-5-yl}methyl)thiophene- 2-carboxamide (BAY 59-7939): An oral, direct factor Xa inhibitor. J. Med. Chem..

[5] Laux V., Perzborn E., Heitmeier S., von Degenfeld G., Dittrich-Wengenroth E., Buchmüller A., Gerdes C., Misselwitz F. (2009). Direct inhibitors of coagulation proteins—The end of the heparin and low-molecular-weight heparin era for anticoagulant therapy?. Thromb. Haemost..

[6] Rose D.K., Bar B. (2018). Direct Oral Anticoagulant Agents: Pharmacologic Profile, Indications, Coagulation Monitoring, and Reversal Agents. J. Stroke Cerebrovasc. Dis..

[7] Fung L.S., Klockau C. (2010). Effects of age and weight-based dosing of enoxaparin on anti-factor xa levels in pediatric patients. J. Pediatr. Pharmacol. Ther..

[8] Streif W., Mitchell L.G., Andrew M. (1999). Antithrombotic therapy in children. Curr. Opin. Pediatr..

[9] Sikes L., Charles K., Antigua A., Patel R., Imboywa S., Cherian P. (2023). Anti-Factor Xa Level Monitoring for Enoxaparin Prophylaxis and Treatment in High-Risk Patient Groups. HCA Healthc. J. Med..

[10] Hanslik A., Kitzmüller E., Tran U.S., Thom K., Karapetian H., Prutsch N., Voitl J., Michel-Behnke I., Newall F., Male C. (2015). Monitoring unfractionated heparin in children: A parallel-cohort randomized controlled trial comparing 2 dose protocols. Blood.

[11] Hirsh J., Anand S.S., Halperin J.L., Fuster V. (2001). Guide to Anticoagulant Therapy: Heparin: A Statement for Healthcare Professionals From the American Heart Association. Arterioscler. Thromb. Vasc. Biol..

[12] Marshall A.M., Trussell T.M., Yee A.M., Malone M.P. (2021). Anti-Xa levels in critically ill children receiving enoxaparin for venothromboembolism prophylaxis. Thromb. Res..

[13] Obuljen J., Jakovljević G., Margetić S., Linarić I., Leniček Krleža J. (2020). Results of anti-Xa activities for monitoring therapeutic and prophylactic doses of low molecular weight heparin in children. Res. Pract. Thromb. Haemost..

[14] Koury J., Hellinga R., Rose J., Abraham S., Subbaswamy A. (2021). Evaluation of Anticoagulant Monitoring in Pediatric Patients Receiving Enoxaparin for Treatment of Venous Thrombosis. J. Pediatr. Pharmacol. Ther..

[15] Rosendaal F.R., Cannegieter S.C., van der Meer F.J., Briët E. (1993). A method to determine the optimal intensity of oral anticoagulant therapy. Thromb. Haemost..

[16] Leung M., Ho S.H., Hamilton D.P., Wu J.K., Dix D.B., Wadsworth L.D., Ensom M.H. (2005). Utility of anti-xa monitoring in children receiving enoxaparin for therapeutic anticoagulation. J. Pediatr. Pharmacol. Ther..

[17] Massicotte P., Adams M., Marzinotto V., Brooker L.A., Andrew M. (1996). Low-molecular-weight heparin in pediatric patients with thrombotic disease: A dose finding study. J. Pediatr..

[18] Punzalan R.C., Hillery C.A., Montgomery R.R., Scott C.A., Gill J.C. (2000). Low-molecular-weight heparin in thrombotic disease in children and adolescents. J. Pediatr. Hematol. Oncol..

[19] Dix D., Andrew M., Marzinotto V., Charpentier K., Bridge S., Monagle P., deVeber G., Leaker M., Chan A.K., Massicotte M.P. (2000). The use of low molecular weight heparin in pediatric patients: A prospective cohort study. J. Pediatr..

[20] Klaassen I.L.M., Sol J.J., Suijker M.H., Fijnvandraat K., van de Wetering M.D., Heleen van Ommen C. (2019). Are low-molecular-weight heparins safe and effective in children? A systematic review. Blood Rev..

[21] Andrade-Campos M.M., Montes-Limón A.E., Fernandez-Mosteirin N., Salvador-Osuna C., Torres M., Lucia-Cuesta J.F., Rubio-Felix D. (2013). Dosing and monitoring of enoxaparin therapy in children: Experience in a tertiary care hospital. Blood Coagul. Fibrinolysis.

[22] Ho S.H., Wu J.K., Hamilton D.P., Dix D.B., Wadsworth L.D. (2004). An assessment of published pediatric dosage guidelines for enoxaparin: A retrospective review. J. Pediatr. Hematol. Oncol..

[23] Greene L.A., Law C., Jung M., Walton S., Ignjatovic V., Monagle P., Raffini L.J. (2014). Lack of anti-factor Xa assay standardization results in significant low molecular weight heparin (enoxaparin) dose variation in neonates and children. J. Thromb. Haemost..

[24] Ignjatovic V., Najid S., Newall F., Summerhayes R., Monagle P. (2010). Dosing and monitoring of enoxaparin (Low molecular weight heparin) therapy in children. Br. J. Haematol..

[25] van Ommen C.H., Luijnenburg S.E. (2024). Anticoagulation of pediatric patients with venous thromboembolism in 2023. Thromb. Res..

[26] Schaefer B., Hausfeld A., Martin M., Steele P., Martin J., Reher S.R., Lane A., Luchtman-Jones L. (2020). Impact of exogenous antithrombin on low molecular weight heparin anti-Xa activity assays in a pediatric and young adult leukemia and lymphoma cohort with variable antithrombin levels. Pediatr. Blood Cancer.

[27] Hashem H., Zeineddin M., Bater R., Amayiri N., Al Qasem W., Hammo B., Sultan I., AlMasri R., Abdel-Razeq H. (2021). Thrombosis and Anticoagulant Therapy Among Pediatric Cancer Patients: Real-Life Data. Cureus.

[28] Barg A.A., Kenet G. (2022). Cancer associated thrombosis in pediatric patients. Best Pract. Res. Clin. Haematol..

[29] Escobar A., Salem A.M., Dickson K., Johnson T.N., Burk K.J., Bashoura L., Faiz S.A. (2022). Anticoagulation and bleeding in the cancer patient. Support Care Cancer.

